# Review of the role and potential clinical value of m6A methylation modifications in the biological process of osteosarcoma

**DOI:** 10.3389/fgene.2025.1522622

**Published:** 2025-03-19

**Authors:** Huaqiang Zhou, Rongbing Shu, Jianming Wu, Jiangjun Zhou, Zhuanyi Yu, Qiuxin Cheng, Zhihao Peng, Min Zhao

**Affiliations:** ^1^ Department of orthopaedic surgery, Yingtan People’s Hospital, YingTan, China; ^2^ Department of Orthopedic, The 908Th Hospital of Joint Logistic Support Force of PLA, Nanchang, China

**Keywords:** m6A modification, osteosarcoma, RNA methylation, epigenetics, writer, eraser, reader

## Abstract

Osteosarcoma (OS), an aggressive bone tumor, is a substantial threat to the quality of life and survival of affected individuals. Despite recent improvements in OS therapies, the considerable variability and chemotherapy resistance of this cancer necessitate continuous research to discover new treatment targets and biomarkers. Recent epigenetic advances highlight the crucial role of N6-methyladenosine (m6A) methylation in cancer. In OS, m6A methylation has been demonstrated to be a pivotal component in the pathogenesis. This review introduces new findings regarding the association between m6A methylation regulators and OS, and summarizes the potential clinical applications of OS and m6A methylation regulators, including the role of m6A methylation in OS proliferation, growth, apoptosis, and cell migration, invasion, and metastasis; relationship between m6A methylation and OS chemotherapy resistance; and relationship between m6A methylation and OS prognosis. Our review had certain limitations. The interaction between m6A methylation regulators and other oncogenic factors, such as lncRNAs and ncRNAs, is not fully understood. We hope that these potential methods will be translated into clinical applications and effective treatment.

## 1 Introduction

Smrke et al. (2021) Osteosarcoma is rare cancer but the most prevalent primary malignant bone tumor, with a direct or indirect origin from stromal cells that traverse cartilage to form neoplastic bone and bone tissue (Beird et al., 2022; Mirabello et al., 2009). With a total incidence of 3.1 per million, this condition is more prevalent in men than in women. There is a bimodal distribution in the age of onset, with the condition most commonly manifested in adolescents (Gill et al., 2013). The distal femur, proximal tibia, and proximal humerus are the three bones with the highest incidence (Marchandet et al., 2021). The primary treatment strategy for osteosarcoma involves preoperative chemotherapy combined with surgical removal of the tumor, followed by additional chemotherapy (Harrison et al., 2018). Patients with limited osteosarcoma have a 7% 5-year survival rate (Meazza and Scanagatta, 2016). The prognosis for patients with osteosarcoma is poor if metastases occur, with survival rates as low as 20%–30% after 5 years. However, patients with osteosarcoma are particularly prone to lung metastasis, the most common location of metastases. To understand osteosarcoma development, metastasis, and prognosis, a deeper understanding of the fundamental molecular processes is required.

N6-methyladenosine (m6A) regulatory factors are associated with a variety of diseases, such as (Xu et al., 2024) rheumatoid arthritis (Zhang N. et al., 2023), glioma (Li et al., 2023), lung cancer (Li et al., 2021), and hepatocellular carcinoma. Similarly, m6A methylation plays a crucial role in the biological process of osteosarcoma. Therefore, the aim of this review is to discuss the involvement of m6A methylation in the biological process and clinical prognosis of osteosarcoma, as well as the potential of m6A regulatory factors in clinical applications.

**FIGURE 1:**
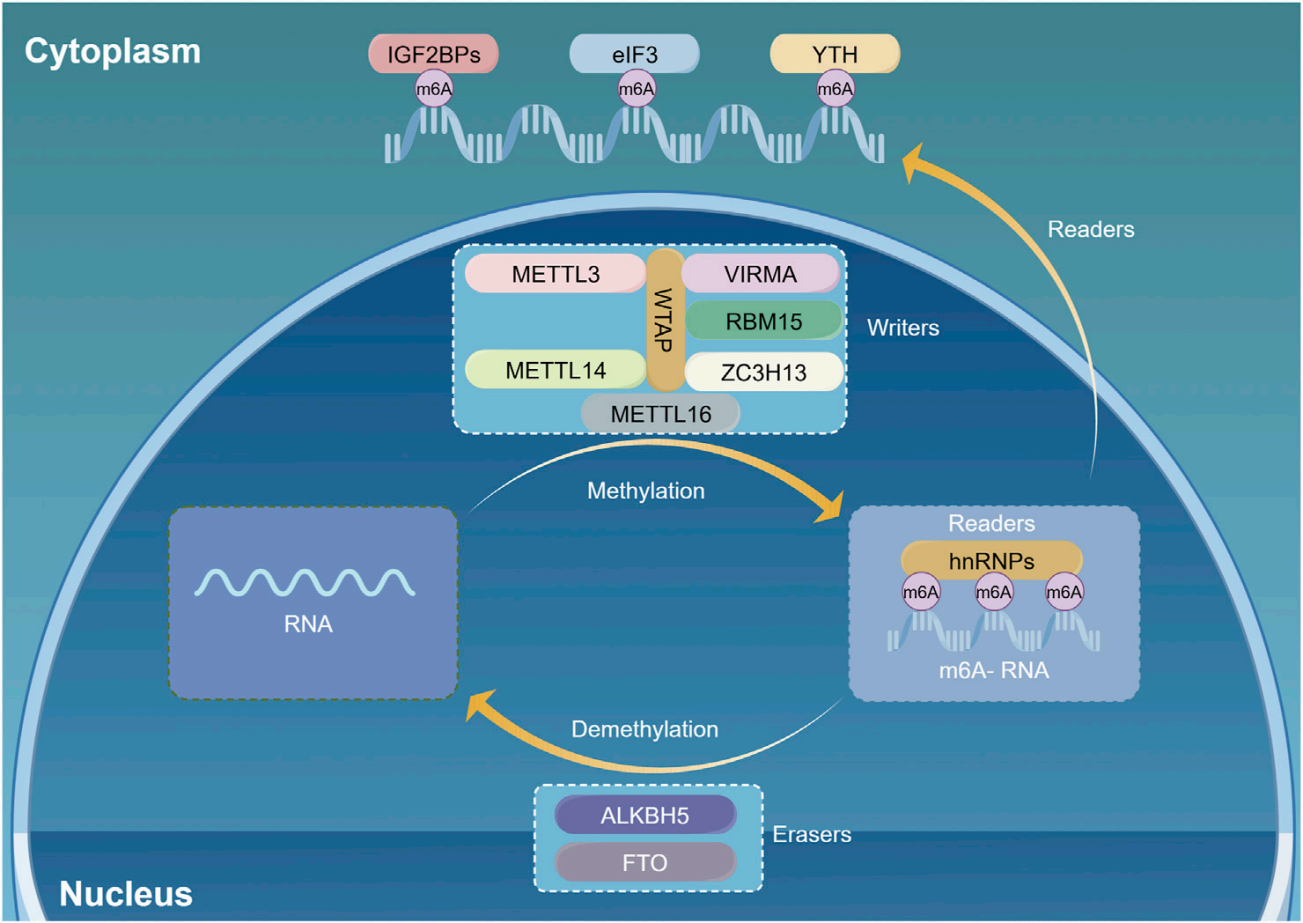
N6-methyladenosine (m6A) methylation dynamically regulates mRNA through multiple pathways. M6A regulatory proteins include m6A methyltransferases (m6A writers), m6A demethylases (m6A erasers), and m6A recognition proteins (m6A readers). The writers act as methyltransferase. The erasers mediate demethylation modifications. M6A readers recognize information about mRNA methylation and are involved in downstream mRNA translation, degradation, and other behavior. (By Figdraw).

One of the most prevalent methylation modifications in eukaryotic mRNA is m6A methylation, which entails the addition or removal of a methyl group to the nitrogen atom located at the sixth position of adenosine (Desrosiers et al., 1974). Its initial identification dates back to 1974 (Dominissini et al., 2012; Meyer et al., 2012). The m6A methylation modification usually happens at the termination codon and the 3ʹ untranslated region, with most of its core sequences being RRACH motifs (R = G/A, H = A/C/U) (Niu et al., 2013). An m6A alteration controls gene expression within the transcriptome, influencing the splicing, transport, degradation, and translation processes of pre-mRNA (Jia et al., 2013). The m6A modification is a dynamic and reversible process (Du J et al., 2020), regulated by various m6A methylation factors such as m6A methyltransferases, demethylases, and methylation-binding proteins (Figure 1).

## 2 Rgulation of m6A methylation

(Meyer et al., 2012) The m6A methyltransferases, also known as writers, are primarily responsible for catalyzing m6A methylation modifications. They are enzyme complexes with multiple components, including methyltransferase-like 3 (METTL3) and 14 (METTL14) (Ping et al., 2014). Furthermore, proteins such as Wilms tumor one associated protein (WTAP) (Wen et al., 2018), zinc finger CCCH-type containing protein 13 (ZC3H13) (Pendleton et al., 2017), methyltransferase-like 16 (METTL16) (Qian et al., 2019), viral-like m6A methyltransferase-associated protein (VIRMA, also known as KIAA1429) (Deng et al., 2018), RNA binding motif protein 15 (RBM15) (Jiang et al., 2021), and CBL proto-oncogene-like protein −1 (CBLL1) are included (Bokar et al., 1997). METTL3, the initially discovered m6A methyltransferase, is the key component of the methyltransferase complex (MTC) (Bokar et al., 1994; Bujnicki et al., 2002; Wang et al., 2016). However, the absence of METTL14 from the complex results in the complete loss of enzymatic activity (Liu et al., 2014; Wang et al., 2016). METTL14 can form a stable 1:1 complex with METTL3, which plays a crucial role in substrate identification (Wang et al., 2016). Whereas METTL3 represents the active peroxidase portion, METTL14 serves to enhance METTL3 activity, stabilize the structure of the complex and promote substrate binding (Ping et al., 2014; Schwartz et al., 2014). WTAP has the capacity to bind to the METTL3-METTL14 complex, thereby localizing it to nuclear speckles and promoting its catalytic activity (Yue et al., 2018). VIRMA proteins are involved in the methylation of adenine at the sixth position of RNA bases, particularly near the stop codon and within the 3ʹ-untranslated region (Wen et al., 2018). ZC3H13 serves to anchor WTAP in the nucleus, thereby facilitating the process of m6A methylation modification (Hsu et al., 2022). Low oxygen levels trigger nuclear m6A modification through METTL4, contributing to hypoxia-driven epithelial-mesenchymal transition and cancer spread (Warda et al., 2017). METTL16 can interact with U6 snRNAs and various other (non-coding RNAs (ncRNAs), as well as numerous lncRNAs and pre-mRNAs. Furthermore, it catalyzes N6-U6 snRNA methylation.

The m6A demethylases, as well as designated as “erasers,” remove the m6A methylation (Jia et al., 2011). Erasers include Erasers include the protein linked to fat mass and obesity (FTO) (Zheng et al., 2013), and AlkB homolog 5 (ALKB5) (Jia et al., 2011). The presence of erasers is responsible for the dynamically reversible process of m6A methylation being. FTO was the first m6A demethylase discovered and is partly situated in nuclear speckles (Fu et al., 2013). Oxidation of m6A by FTO produces two intermediates: hm6A (N6-hydroxymethyladenosine) and f6A (N6-formyladenosine). These intermediates demethylate m6A. ALKBH5 is the second identified m6A demethylase that removes m6A marks in both living organisms and laboratory settings (Zheng et al., 2013). This influences mRNA export, RNA metabolism, and the formation of mRNA processing complexes in nuclear speckles.

Zhu et al. (2014) The m6A methyl-reading protein, also designated a reader, is capable of recognizing m6A methylation modifications and subsequently binding to them, thereby exerting an influence on mRNA stability, metabolism and translation. Additionally, it is essential to incorporate the YT521-B homology (YTH) domain group (Shi et al., 2019), along with heterogeneous nuclear ribonucleoproteins (HNRNPA2B1, HNRNPC, and HNRNPA1) (Meyer and Jaffrey, 2017), and eukaryotic initiation factor 3 (EIF3) (Pan et al., 2021). ELAVL1 and (Huang et al., 2018) IGF2BPs proteins participate in this process (Huang et al., 2018). YTHDF1 associates with promoters to promote mRNA translation and protein synthesis (Wang et al., 2014). YTHDF2 binds selectively to m6A-containing mRNAs, whereas its amino terminus localizes to cellular RNA decay sites, allowing it to function as a mRNA decay inhibitor (Shi et al., 2017). YTHDF3 enhances protein production in collaboration with YTHDF1, and influences YTHDF2, which can impact the degradation of methylated mRNA (Roundtree et al., 2017). Nuclear export and splicing of mRNA are mediated by YTHDC1 (Hsu et al., 2017). YTHDC2 boosts translation efficiency and concurrently lowers the mRNA levels of its targets (Li et al., 2019; Muller et al., 2019; Wang et al., 2019). IGF2BP enhances mRNA stability and expression (Meyer et al., 2015). A single 5ʹ-UTR m6A can directly bind to eukaryotic initiation factor 3 (eIF3), enabling translation during stress by circumventing 5ʹ-cap-binding proteins (Alarcon et al., 2015). HNRNPA2B1 binds to specific mRNAs, aiding in the initial processing of primary miRNAs through a mechanism that includes the microRNA microprocessor complex protein DGCR8 (Liu et al., 2015). HNRNPC is involved in the maturation of pre-mRNA (Cai et al., 2022). An m6A-dependent function of ELAVL1 facilitate tumor initiation and progression.

## 3 The impact of m6A methylation changes on osteosarcoma

**FIGURE 2:**
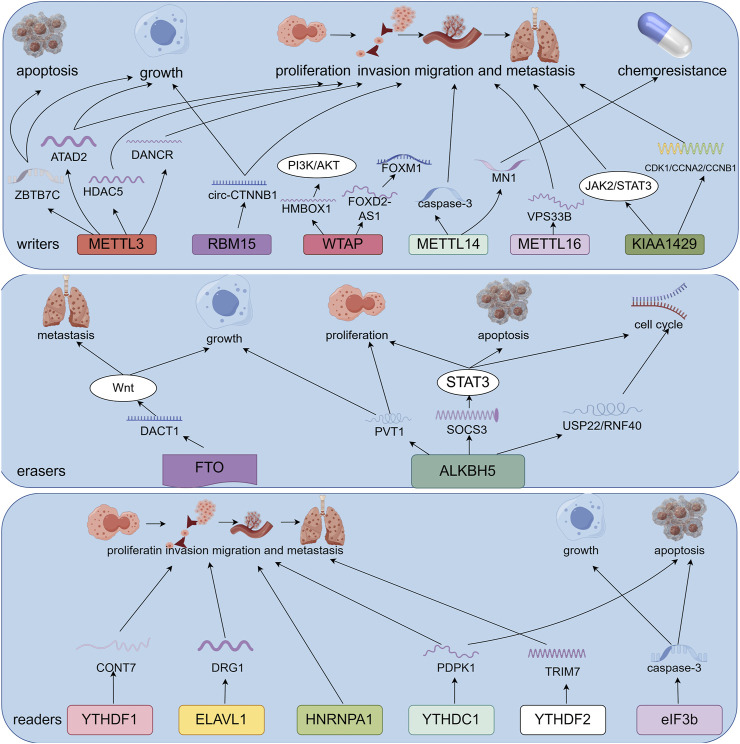
Potential role of m6A RNA modification in osteosarcoma. M6A regulates the expression of downstream genes, which contributes to osteosarcoma development, including apoptosis, cell proliferation, chemotherapy resistance, invasion, metastasis, and migration (By Figdraw).

M6A methylation influences the growth, spread, invasion, and programmed cell death of osteosarcoma cells. This review provides an overview of various m6A regulators involved in biological processes associated with osteosarcoma. (Figure 2; Table 1).

**TABLE 1:** Roles of m6A regulators in osteosarcoma.

m6A regulators	Function	Targeted genes/pathway	Biological function	Vitro/vivo	References
METTL3	Writers	HDAC5/miR-142-5p/ARMC8	proliferation	Vitro and Vivo	Jiang et al. (2022)
METTL3	Writers	ZBTB7C	Growth apoptosis	Vitro and Vivo	An et al. (2023)
METTL3	Writers	ATAD2 Bcl-2/Bax axis and Caspase cascade	proliferation migration invasion apoptosis colony formation	Vitro	Zhou L. et al. (2020)
METTL3	Writers	DANCR	proliferation migration invasion	Vitro	Zhou et al. (2023)
METTL14	Writers	MN1	proliferation colony formation migration invasion	Vitro and Vivo	Li et al. (2022)
METTL14	Writers	Caspase-3	proliferation apoptosis migration invasion	Vitro	Liu et al. (2020)
METTL16	Writers	VPS33B/PI3K/Akt	proliferation migration invasion	Vitro	Cheng et al. (2023)
WTAP	Writers	PI3K/Akt	proliferation growth metastasis	Vitro	(Chen et al., 2020b)
WTAP	Writers	FOXD2-AS1	proliferation growth	Vitro and Vivo	Ren Z. et al. (2022)
KIAA1429	Writers	JAK2/STAT3	proliferation growth migration invasion	Vitro and Vivo	Luo et al. (2023)
KIAA1429	Writers	—	proliferation apoptosis colony formation	Vitro and Vivo	Sun et al. (2024)
RBM15	Writers	Circ-CTNNB1	proliferation invasion	Vitro and Vivo	Yang et al. (2023)
FTO	Erasers	DACT1/Wnt	proliferation colony formation growth apoptosis metastasis migration invasion	Vitro and Vivo	Lv et al. (2022)
ALKBH5	Erasers	STAT3	proliferation growth apoptosis migration cell cycle arrest	Vitro and Vivo	Yang et al. (2022)
ALKBH5	Erasers	PVT1	proliferation growth	Vitro and Vivo	Chen et al. (2020b)
YTHDC1	Readers	PDPK1	proliferation apoptosis	Vitro	Cao et al. (2022)
YTHDF1	Readers	CONT7	proliferation migration invasion	Vitro and Vivo	Wei et al. (2022)
YTHDF2	Readers	TRIM7	migration invasion	Vitro and Vivo	Zhou C. et al. (2020)
YTHDF3	Readers	PGK1	proliferation growth	Vitro and Vivo	Liu et al. (2023)
eIF3b	Readers	Caspase-3 cleavage/PARP	growth apoptosis	Vitro	Choi et al. (2017)
eIF3H	Readers	—	proliferation cell cycle arrest apoptosis colony formation	Vitro and Vivo	Hong et al. (2018)
eIF3C	Readers	SAPK/JNK	proliferation apoptosis colony formation	Vitro	Gao et al. (2019)
ELAVL1	Readers	DRG1	migration colony formation apoptosis	Vitro	Ling et al. (2020)
LRPPRC	Readers	FOXM1	cell cycle arrest invasion colony formation	Vitro	Zhao et al. (2023)
HNRNPA1	Readers	—	proliferation migration invasion	Vitro	Fu et al. (2021)

### 3.1 The role of m6A methylation in the proliferation, growth and apoptosis of osteosarcoma

Evan and Vousden (2001) The inhibition of cell proliferation and cell death together promote the development of cancer. Jiang et al. (2022) reported that overexpression of METTL3 enhanced osteosarcoma cell proliferation, whereas METTL3 silencing significantly reduced osteosarcoma cell proliferation. METTL3 silencing leads to the inhibition of HDAC5 expression, which can reduce the expression of miR-142-5p, which targets ARMC8. *In vivo* experiments, confirmed that METTL3 silencing inhibited *in vivo* osteosarcoma proliferation and reduced tumor volume and weight. These results indicated that METTL3 silencing suppressed osteosarcoma proliferation *in vivo* via the HDAC5/miR-142-5p/ARMC8 axis. An et al. (2023) demonstrated that METTL3 distortion drives osteosarcoma progression by promoting osteosarcoma growth and preventing osteosarcoma cell apoptosis through ZBTB7C m6A modification and sustained ZBTB7C activation. CCK8 and live-dead staining experiments confirmed that STM2457 induced cell death in MNNG/HOS cells. In the *in vivo* experiments, STM2457 treatment significantly reduced tumor growth, indicated by a decrease in tumor volume and weight. Zhou L. et al. (2020) demonstrated that METTL3 is an oncogene in the progression of osteosarcoma; when METTL3 is silenced, the percentage of apoptosis in tumor cells significantly increases. Western blotting showed that silencing METTL3 promoted apoptosis in osteosarcoma cells by regulating the Bcl-2/Bax axis and Caspase cascade. METTL14 enhances the catalytic activity of METTL3 by binding to METTL3. Liu et al. (2020) found that the overexpression of METTL14 can significantly inhibit proliferation. In addition, METTL14 induces tumor cell apoptosis by activating caspase-3. WTAP plays a significant role in RNA metabolism by WTAP, which binds to the METTL3-METTL14 complex. Ren Z. et al. (2022) found that WTAP enhanced the stability of FOXD2-AS1, an RNA associated with poor prognosis in osteosarcoma. FOXD2-AS1 overexpression increased the proliferation of MG63 cells *in vitro*, and FOXD2-AS1 knockdown decreased the proliferation of Saos-2 cells. FOXD2-AS1 overexpression promoted tumor growth in subcutaneously injected mice *in vivo*. Luo et al. (2023) reported that KIAA1429 is upregulated in osteosarcoma and promotes the malignant progression of osteosarcoma cells through the JAK2/STAT3 pathway. Deletion reduces cell proliferation. KIAA1429 knockdown significantly reduced tumor volume and weight *in vivo*. Similarly, Sun et al. (2024) observed that silencing KIAA1429 decreased m6A methylation levels, inhibited cell proliferation and colony-forming ability, prevented tumor growth *in vivo* and promoted osteosarcoma cell apoptosis. Lv et al. (2022) found that FTO is highly expressed in the osteosarcoma. Downregulation of FTO in osteosarcoma cells significantly reduce cell proliferation and colony formation ability, and increase apoptosis. However, the exogenous expression of FTO reversed these results. Yang et al. (2022) suggest that overexpression of ALKBH5 leads to decreased m6A methylation, inactivation of the STAT3 pathway, inhibition of cell proliferation, and promotion of apoptosis by inducing G0/G1 arrest, In an *in vivo* experiment, tumor growth was effectively inhibited when ALKBH5 overexpressing U2OS cells were subcutaneously transplanted into nude mice. Yadav et al. (2022) demonstrated that ALKBH5 mediated m6A deficiency in osteosarcoma leads to increased expression of the histone deubiquitinase USP22 and ubiquitin ligase RNF40, resulting in the inhibition of histone H2A monoubiquitination and induction of key tumor promoting genes, thereby driving uncontrolled cell cycle progression, continuous replication, and DNA repair. When ALKBH5 was silenced, apoptosis of osteosarcoma cells increased. Zhou C. et al. (2020) revealed that ALKBH5 can bind to PVT1 and inhibit its degradation, reducing the m6A modification of PVT1 and thereby inhibiting the binding of YTHDF2 to PVT1. ALKBH5-mediated upregulation of PVT1 promotes osteosarcoma cell proliferation *in vitro* and tumor growth *in vivo*. Liao et al. (2022) Most stages of m6A-modified RNA metabolism are regulated by YTH proteins, including splicing, exporting, and translating precursor mRNA. Cao et al. (2022) reported that YTHDC1 promotes the malignant progression of osteosarcoma cells via the m6A methylation of PDPK1 mRNA. YTHDC1 knockdown inhibits proliferation and promotes apoptosis in osteosarcoma cells. Liu et al. (2023) found that YTHDF3 directly binds to PGK1 and enhances its stability, thereby promoting cell proliferation and colony formation by promoting aerobic glycolysis in osteosarcoma cells. Choi et al. (2017) confirmed that U2OS cells experienced significant inhibition of cell growth and increased apoptosis when eIF3b was silenced. Western blotting showed that eIF3b silencing increased PARP and caspase-3 cleavage, which is a readout of an active apoptotic cellular program. Hong et al. (2018) observed that the knockdown of EIF3H inhibited the proliferation and colony formation of Saos-2 and U2OS cells. Flow cytometry showed that inhibition of EIF3H expression induced G0/G1 phase arrest and promote apoptosis in osteosarcoma cells. *In vivo* experiments showed that Saos-2 cells infected with shEIF3H developed significantly smaller tumors and reduced tumors weight in mice. Gao et al. (2019) found that high EIF3C mRNA expression levels in osteosarcoma tissues. Proliferation and colony formation ability were significantly reduced in EIF3C-knocked down U-2OS cells when compared with those in controls. The SAPK/JNK signaling pathway was significantly enhanced in U-2OS cells treated with shEIF3C, and apoptosis was observed in 16.39% of U-2OS cells treated with shEIF3C lentivirus, indicating that EIF3C promoted cell apoptosis and inhibited tumor proliferation through the SAPK/JNK pathway. Fu et al. (2021) proved that HNRNPA1 is downstream of FASN and acts as a biomarker and oncogene in osteosarcoma. Silencing HNRNPA1 led to a decrease in the proliferation ability and colony number of the osteosarcoma cell lines 143B and HOS. In summary, these results indicate that m6A is crucial for the proliferation, growth and apoptosis of osteosarcoma cells *in vitro* and *in vivo*.

### 3.2 The role of m6A methylation in the migration, invasion and metastasis of osteosarcoma cells

Yang et al. (2021) The migration and invasion of tumor cells are the key factors of tumor progression and metastasis. Tumor metastasis remains the most common cause of cancer-related deaths. METTL3 is associated with the migration, invasion, and metastasis of osteosarcoma cells. Zhou et al. (2023) observed that METTL3 expression is elevated in all osteosarcoma cell lines; and promoted osteosarcoma cell proliferation, migration, and invasion by increasing DANCR mRNA stability via m6A modification. Zhou L. et al. (2020) demonstrated that METTL3 is an oncogene in the progression of osteosarcoma. When METTL3 was silenced, the vitality, migration, invasion ability, and cell colony-forming abilities were also suppressed in MG63 and SAOS-2 cells. Li et al. (2022) reported that METTL14 is highly expressed in osteosarcoma specimens and promotes the proliferation and metastasis of osteosarcoma *in vitro* and *in vivo*. METTL14 knock down inhibits metastasis. They also found that MN1 is a downstream target of METTL14, and knockdown of MN1 significantly inhibited the proliferation, colony formation, migration and invasion of U2OS and 143B cells. However, Liu et al. (2020) found that patients with high METTL14 expression have higher early survival rates. METTL14 overexpression significantly inhibits the migration and invasion of osteosarcoma cells. Cheng et al. (2023) demonstrated that METTL16 can downregulate VPS33B in an m6A-dependent manner, and the METTL16/VPS33B pathway can promote the proliferation, migration and invasion of osteosarcoma by activating the PI3K/Akt pathway. Chen et al. (2020a) demonstrated that WTAP suppressed HMBOX1 expression through an m6A-dependent process, subsequently promoting osteosarcoma proliferation, growth and metastasis via the PI3K/AKT signaling pathway. Luo et al. (2023) found that KIAA1429 knockdown significantly downregulated the migration and invasion of osteosarcoma cells. Yang et al. (2023) reported that the expression of Circ-CTNNB1 is relatively high in osteosarcoma tissues and cells, which can directly interact with RBM15. Silencing Circ-CTNNB1 inhibited the growth and invasiveness of osteosarcoma cells both *in vitro* and *in vivo*. By contrast, Circ-CTNNB1 overexpression induced an increase glucose uptake, lactate production, ATP levels, proliferation, and invasiveness of osteosarcoma cells *in vitro* and *in vivo*. These results suggest that Circ-CTNNB1 promotes the malignant progression of osteosarcoma cells through aerobic glycolysis. Lv et al. (2022) demonstrated that FTO promotes the growth and metastasis of osteosarcoma *in vitro* and *in vivo* through the DACT1/Wnt signaling axis. After FTO knock down, the migration and invasion of osteosarcoma cells were significantly inhibited. In the *in vivo* experiments, when FTO was downregulated, the volume and weight of the in-situ tumors were significantly reduced compared with those in the control group, and the number of lung metastatic nodules was also reduced. However, the exogenous expression of FTO reversed these results. Yadav et al. (2022) reported that ALKBH5 is highly expressed in osteosarcoma and promotes its growth and progression. ALKBH5 silencing significantly reduced the migratory ability of osteosarcoma cells *in vivo*. Wei et al. (2022) found through Transwell experiments that inhibition of YTHDF1 expression suppressed the proliferation, migration, and invasion of osteosarcoma cells. In vivo experiments, the downregulation of YTHDF inhibited the growth of osteosarcoma tumors. We found that CONT7 may be a downstream gene. Zhou C. et al. (2020) identified that YTHDF2 promotes the m6A modification of TRIM7 to regulate the malignant progression of osteosarcoma. Through *in vitro* Transwell analysis, we found that TRIM7 knockdown inhibited the migration and invasion of osteosarcoma cells. In vivo experiments, the number of lung metastatic nodules and instances of metastasis in mice were significantly decreased by TRIM7 knockdown. Ling et al. (2020) found that DRG1 silencing decreased osteosarcoma cell viability, inhibited migration and colony formation abilities, led to cell cycle arrest in the G2/M phase and induced apoptosis. METTL3 and ELAVL1 knockdown impairs DRG1 stability. These results indicate that DRG1 plays a tumorigenic role in osteosarcoma, and ELAVL1 and METTL3 induce the upregulation of DRG1 in osteosarcoma in an m6A dependent manner. Zhao et al. (2023) observed that LRPPRC may participate in promoting stemness and regulating tumor progression in osteosarcoma through FOXM1. LRPPRC knockdown significantly increased the proportion of the G1/G0 phase in osteosarcoma cells, significantly reduced their invasion and tumor formation ability, as well as ATP synthesis and mitochondrial DNA content in osteosarcoma cells. These results suggest that LRPPRC regulate tumor occurrence and progression by modulating mitochondrial function. Fu et al. (2021) revealed that HNRNPA1 silencing decreased the migration ability of 143B and HOS cells. In summary, these findings indicated that m6A regulatory factors play important role in the migration, invasion, and metastasis of osteosarcoma cells.

## 4 Methylation of m6A in osteosarcoma: potential clinical applications

Increasing evidence suggests that m6A regulators are associated with better clinical outcomes in patients with osteosarcoma. In individuals with osteosarcoma, abnormal levels of m6A-related regulators are closely linked to an unfavorable prognosis and resistance to chemotherapy. M6A modifications may serve as promising biomarkers or therapeutic targets in osteosarcoma (Table 2).

**TABLE 2:** Potential clinical applications of m6A methylation in osteosarcoma.

Source	Non-tumor samples	Tumor samples	M6A regulators	Role	Potential application	References
Clinical samples	0	120	METTL14/IGF2BP2	chemoresistance	biomarker/therapeutic target	Li et al. (2022)
Clinical samples	20	100	METTL3/14/YTHDF2	Metastasis/chemoresistance	biomarker	Zhou C. et al. (2020)
Clinical samples	40	40	METTL3	worse prognosis	therapeutic target	Zhou et al. (2023)
Publicly datasets	0	88	RBM15/METTL3/LRPPRC	worse prognosis	biomarker	Huang et al. (2022)
TMA cohort	65	120	METTL3/METTL14/YTHDF2/KIAA1429/HNRNPA2B1	worse prognosis	biomarker/therapeutic target	(J. Li et al., 2020)
Clinical samples	50	50	METTL3	worse prognosis	therapeutic target	Jiang et al. (2022)
RT-qPCR/microarray/RNA sequencing data	71	250	KIAA1429	Oncogene	biomarker/therapeutic target	Sun et al. (2024)
Publicly datasets	396	88	RBM15/YTHDC1	worse prognosis	biomarker	Jiang et al. (2023)
Clinical samples	0	82	ALKBH5/YTHDF2	worse prognosis	biomarker/therapeutic target	Yang et al. (2022)
Publicly datasets	3	44	WTAP	worse prognosis	therapeutic target	Chen et al. (2020a)

### 4.1 Methylation of m6A contributes to chemotherapy resistance in osteosarcoma

Chang et al. (2021) The most frequent approach for treating advanced tumors involves a combination of surgical procedures and radiation therapy (Steinbichler et al., 2018). Nevertheless, the development of resistance to chemotherapeutic agents has the potential to result in tumor recurrence and treatment failure. Li et al. (2022) found that tumor growth and weight in MN1 and METTL14-knockdown groups treated with ATRA were significantly reduced in tumor xenografts. Tumor growth and weight in the group overexpressing MN1 in METTL14 knockdown cells treated with ATRA were not significantly reduced. Expression levels of MN1 and Ki-67 in growth-damaged tumors generated by METTL14-knockdown in osteosarcoma cells were lower than those in the control group. These results indicate that METTL14 upregulates MN1 to induce ATRA resistance in osteosarcoma cells. He et al. (2022) observed that the knockout of IGF2BP1 significantly inhibited the proliferation of MG-63/Dox and HOS/Dox cells, enhancing their sensitivity to Dox chemotherapy. In vivo experiments, IGF2BP1 increased Dox sensitivity and reduced tumor volume and weight in mice. These findings suggest that IGF2BP1 regulates Dox resistance in osteosarcoma. Zhou C. et al. (2020) showed that MG63 cells with TRIM7 knockdown were more sensitive to ADR and MTX treatment through CCK-8 assay. In vivo experiments, patient-derived xenograft (PDX) mice receiving ADR or MTX chemotherapy with high TRIM7 expression exhibited larger tumors than those in PDX mice with low TRIM7 expression. This indicates that TRIM7 plays an important role in the regulation of chemotherapy sensitivity in osteosarcoma. Based on the findings of study, m6A methylation regulators may be involved in osteosarcoma chemoresistance. Additionally, these regulators can potentially reverse osteosarcoma resistance.

### 4.2 The presence of m6A methylation is linked to a worse prognosis for osteosarcoma

An in-depth study of the m6A methylation regulator expression in both osteosarcoma and normal tissues revealed a strong link between several m6A methylation regulators and the outcomes of patients with osteosarcoma. Zhou et al. (2023) reported that Kaplan–Meier curve analyses indicated that patients with high METTL3 levels had a relatively poor prognosis. Huang et al. (2022) identified a significant association between RBM15, METTL3, and LRPPRC and poor survival outcomes in patients with osteosarcoma. Li et al. (2020) found that univariate analysis based on the TMA queue indicated that high expression of KIAA1429 and HNRNPA2B1, as well as low expression of METTL3 and METTL14, were potential independent prognostic risk factors in patients with osteosarcoma. Jiang et al. (2022) assigned 50 patients with osteosarcoma to the METTL3 high expression and low expression group, with the median METTL3 mRNA expression in osteosarcoma tissues as the critical value. Survival analysis indicated that high METTL3 expression indicates a poorer overall survival rate. Sun et al. (2024) conducted a survival analysis based on data from the TARGET database and found that patients with high KIAA1429 expression had shorter overall survival. He et al. (2022) conducted a survival analysis based on TCGA database and found that patients with high IGF2BP1 expression had poor overall survival. Jiang et al. (2023) employed LASSO and multifactorial COX regression analysis to develop a prognostic model for osteosarcoma based on RBM15 and YTHDC1. According to their findings, patients with high-risk conditions had a significantly lower survival rate than those with low-risk conditions. Lv et al. (2022) showed that patients with high FTO expression had a poor 5-year overall survival. Liu et al. (2021) conducted a univariate Cox proportional hazards regression model and found that FTO and IGF2BP2 levels were associated with prognosis. Yang et al. (2022) conducted a Kaplan–Meier survival analysis and showed that the overall survival time of patients with low ALKBH5 expression was significantly shorter than that of patients with high ALKBH5 expression. Chen et al. (2020a) demonstrated that WTAP expression was significantly elevated in osteosarcoma and patients with high WTAP levels had low overall survival. Both single and multi-variate Cox regression analyses revealed that WTAP independently predicted overall survival in patients with osteosarcoma. These studies indicated a strong link between m6A methylation regulators and outcomes in patients with osteosarcoma. Patients with osteosarcoma may benefit from the therapeutic targeting of these regulators, thereby improving overall survival.

## 5 Discussion

Recently, studies have been conducted to determine how m6A methylation influences osteosarcoma development. The findings of this study suggest that m6A plays a crucial role in the spread of osteosarcoma, resistance to treatment, and patient outcomes. The influence of m6A on cell growth, movement, invasion, signaling pathways, programmed cell death, and downstream genes are crucial mechanisms of its action. In addition to osteosarcoma (Chen et al., 2022; Liu et al., 2022; Wu et al., 2018), m6A has been linked to other bone tissue disorders, including osteoporosis, osteoarthritis, and rheumatoid arthritis. This offers insights into potential avenues of research pertaining to the regulation of m6A in bone tissue. In addition to osteosarcoma, m6A has been linked to several different tumor types. Zhu et al. (2022) suppressed Hedgehog signaling through METTL3-induced m6A modification of STEAP2 mRNA, hindering the advancement of thyroid-like carcinoma by preventing epithelial-to-mesenchymal transition (Sun et al., 2023). The ALKBH5 protein triggers FAK signaling by demethylating m6A on ITGB1 mRNA, leading to lymphangiogenesis and lymph node metastasis in ovarian cancer Chang et al. (2020). Research on individuals with breast cancer indicates that YTHDF3 enhances the translation of genes tagged with the m6A modification, thereby promoting the formation of brain metastases (Wang et al., 2020). Enhanced expression of METTL14 facilitated pancreatic cancer proliferation and spread by elevating m6A methylation on PERP mRNA. These studies have shown that m6A is associated with various cancer types. Furthermore, various studies have identified unique functions of identical m6A methylation regulators in the same type of cancer. Li et al. (2019) found that in colorectal cancer, METTL3 promotes tumor growth through a pathway dependent on m6A-IGF2BP2. However, Deng et al. (2019) found that METTL3 inhibited the growth and movement of colorectal cancer cells through the ERK/p38 signaling pathway. This phenomenon can be attributed to several factors, including the heterogeneity of tumors, diverse origins of tumors, and use of disparate research methodologies and mechanisms. Furthermore. Tian et al. (2023) discovered that MHL stimulated osteoclastogenesis by targeting METTL3 and increasing METTL3 expression. By contrast, Wang et al. (2023) observed that EGR1 upregulates METTL3, which in turn increases CHI3L1 expression and promotes osteoclastogenesis. This suggests that m6A methylation may be a double-edged sword, potentially accelerating or inhibiting tumor progression through different mechanisms.

Li et al. (2022) reported that ATRA treatment could be a promising complementary therapy for patients with osteosarcoma. However Ren L. et al. (2022) propose that ATRA is not a standard first-line chemotherapeutic drug for osteosarcoma. The IC50 value of ATRA killing osteosarcoma cells is very high (>50 μ m), indicating that ATRA may not be an ideal drug for the treatment of osteosarcoma. Lv et al. (2022) found that entacapone has the potential to inhibit osteosarcoma via the FTO/DACT1 axis. In a nude mouse tibia orthotopic model, entacapone inhibited tumor growth in a dose-dependent manner, significantly reduced tumor volume and weight, and reduce the number of lung metastatic nodules. In addition Deng et al. (2022) suggested that AI-assisted technology could be widely used in the discovery and development of drug candidates. Using a series of AI technologies, we can better screen and predict potential compounds, modify and optimize chemical structures, and evaluate the drug formation of lead compounds targeting m6A modification. Unfortunately, we did not retrieve clinical trials on potential therapeutic targets, which may have been related to ethical issues, high costs, and technical limitations. However, clinical research has tseveral limitations and risks. First, several studies have been conducted based on bioinformatic analyses. Although some studies have established risk-assessment models, their accuracy and predictive abilities require further verification. Second,due to the heterogeneity of tumors, different patients may respond differently to treatment, which increases the complexity of research and treatment. Third, different m6A methylation regulators may affect patients’ response to immunotherapy and targeted therapies. Fourth, some studies are only based on database analysis and lacked validation in clinical trials.

It would be beneficial for future studies to provide more reliable evidence to support this hypothesis. Notably, m6A methylation changes provide new perspectives on the molecular processes of osteosarcoma and could aid in the development of more effective treatments. Nevertheless, current research on m6A methylation modifications in osteosarcoma has predominantly focused on METTL3, METTL14, WTAP, ALKBH5, and YTHDF2, with few studies on other m6A methylation regulators. Additionally, research on m6A methylation in the tumor microenvironment is limited. Zhang Y. et al. (2023) conducted a review and found that ncRNAs are key regulators of the osteosarcoma microenvironment. It can regulate the glycolytic process of osteosarcoma, leading to metabolic dysregulation of the tumor microenvironment and participation in tumor immunity (Shi et al., 2024). There is evidence that m6A interactions with non-coding RNAs are linked to the biological processes of many diseases, and cancer development, prognosis, drug resistance, and therapy. Therefore, it is reasonable to posit that m6A and ncRNAs in the tumor microenvironment may represent an avenue for future research.

## 6 Conclusion

In this study, we review the latest advancements in the understanding of the impact of m6A modifications on osteosarcoma. Alterations in m6A methylation are crucial in various essential processes related to osteosarcoma, such as growth, movement, invasion, programmed cell death of osteosarcoma cells, tumor spread, emergence of drug resistance, and patient outcome. As experimental and high-throughput sequencing technologies advance, researchers will be able to identify additional m6A methylation-related to osteosarcoma, thereby revealing more potential diagnostic and prognostic biomarkers for osteosarcoma. This will have significant implications for identifying targets for potential therapeutic intervention and tumor markers of osteosarcoma, as well as for improving the current status of osteosarcoma treatment.
